# Fully automated pipeline for measurement of the thoracic aorta using joint segmentation and localization neural network

**DOI:** 10.1117/1.JMI.10.5.051810

**Published:** 2023-10-31

**Authors:** Sudeep Katakol, Timothy J. Baker, Zhangxing Bian, Yanglong Lu, Greg Spahlinger, Charles R. Hatt, Nicholas S. Burris

**Affiliations:** a University of Michigan, Department of Electrical and Computer Engineering, Ann Arbor, Michigan, United States; b University of Michigan, Department of Radiology, Ann Arbor, Michigan, United States; c Johns Hopkins University, Department of Electrical and Computer Engineering, Baltimore, Maryland, United States; d Imbio Inc., Minneapolis, Minnesota, United States

**Keywords:** aorta, aneurysm, aortic diameter, segmentation, localization

## Abstract

**Purpose:**

Diagnosis and surveillance of thoracic aortic aneurysm (TAA) involves measuring the aortic diameter at various locations along the length of the aorta, often using computed tomography angiography (CTA). Currently, measurements are performed by human raters using specialized software for three-dimensional analysis, a time-consuming process, requiring 15 to 45 min of focused effort. Thus, we aimed to develop a convolutional neural network (CNN)-based algorithm for fully automated and accurate aortic measurements.

**Approach:**

Using 212 CTA scans, we trained a CNN to perform segmentation and localization of key landmarks jointly. Segmentation mask and landmarks are subsequently used to obtain the centerline and cross-sectional diameters of the aorta. Subsequently, a cubic spline is fit to the aortic boundary at the sinuses of Valsalva to avoid errors related inclusions of coronary artery origins. Performance was evaluated on a test set of 60 scans with automated measurements compared against expert manual raters.

**Result:**

Compared to training separate networks for each task, joint training yielded higher accuracy for segmentation, especially at the boundary (p<0.001), but a marginally worse (0.2 to 0.5 mm) accuracy for landmark localization (p<0.001). Mean absolute error between human and automated was ≤1  mm at six of nine standard clinical measurement locations. However, higher errors were noted in the aortic root and arch regions, ranging between 1.4 and 2.2 mm, although agreement of manual raters was also lower in these regions.

**Conclusion:**

Fully automated aortic diameter measurements in TAA are feasible using a CNN-based algorithm. Automated measurements demonstrated low errors that are comparable in magnitude to those with manual raters; however, measurement error was highest in the aortic root and arch.

## 1 Introduction

Thoracic aortic aneurysm (TAA) is a common disease affecting between 1% and 3% of the United States population over the age of 50, which is characterized by enlargement of the largest artery in the body (i.e., the thoracic aorta) as a result of weakening of the aortic wall.^1^ TAA is a largely silent disease until potentially fatal complications, such as rupture, occur. Patients with TAA undergo regular surveillance imaging using computed tomography angiography (CTA) to monitor the overall size of their aneurysm and to detect growth, assessments that in turn determine the need for surgical repair.^2^ Aortic diameter measurements are used to assess TAA, with measurements performed on volumetric CTA imaging data by manual analysts (i.e., technologists or the physicians themselves) using dedicated three-dimensional (3D) image rendering software. Diameter measurements are made in standardized anatomic locations along the length of the aorta to minimize measurement variability and ensure comparability of measurements across time.^3^^,^^4^

Currently, such measurements are performed manually by human raters, either technologists or physicians, using 3D image analysis software. This process is time and labor intensive, requiring between 15 and 45 min of focused effort to generate a complete set of measurements depending on the complexity of pathology. TAAs typically grow at very slow rates, and aortic diameters typically only enlarge by 1 to 3 mm between surveillance imaging exams,^5^ meaning that significant effort is required on the part of the human rater to ensure accurate diagnostic information. The rigorous and repetitive nature of this task can lead to fatigue in human raters, comprising measurement accuracy and speed.

Recent work aims at automating the labor intensive aorta measurement process by employing deep learning^6^^–^^9^ or other^10^ techniques. While promising, some approaches are not fully automated and require manual selection of measurement sites^6^ while others provide measurements at only a subset of the standard clinical locations.^9^^,^^10^ Our goal is to develop a system that measures diameters at all standard clincical locations while improving upon the accuracy and reliability of other fully automated systems.^7^^,^^8^

To overcome limitations of manual and prior automated approaches, we develop a machine learning (ML) based algorithm that allows us to obtain aortic measurements in a fully automated fashion. Our current method builds on recent work involving the use of convolutional neural networks (CNNs) to segment^6^^,^^11^^,^^12^ and localize key anatomic landmarks on the aorta.^13^ Specifically, in this work, we aimed to develop a CNN that simultaneously performs joint aortic segmentation and landmark localization and subsequently use the output of this network to generate an aortic centerline and extract maximal cross-section diameters at points along the centerline corresponding to the standard clinical measurement locations.

## 2 Method

### 2.1 Pipeline

The objective of our automated measurement pipeline is to extract aortic diameter measurements at nine standard aortic locations used clinically.^3^^,^^4^ We achieve this through localization of six anatomically well-defined landmarks, from which three additional measurement locations are automatically derived in a manner that mirrors the manual clinical workflow. These six landmarks are: center of the aortic valve (L1) proximal arch (L2), just distal to the left subclavian artery (L3), celiac artery (L4), mid-arch (L5), and sinotubular junction (STJ) (L6). Three additional measurements at standard locations are derived from these six landmarks including: mid ascending aorta (mid between the STJ and proximal arch), 2 cm distal to the left subclavian artery (along the centerline), and 2 cm proximal to celiac artery (along centerline).

To obtain the aortic measurements from a given CT scan I, we begin by reorienting the CT image and rescaling it to have isotropic unit-spacing. We input this standardized CT image to a CNN that segments the aorta and localizes (L=6) landmarks of interest. Using the landmarks and segmentation, we estimate an aortic centerline as well as cross-sections along it. The diameters of the cross-section closest to desired locations are output.

### 2.2 Dataset and Training Details

We retrospectively collected 212 CTA scans from 67 patients with a diagnosis of TAA undergoing routine clinical imaging surveillance at our center. Standard clinical image acquisition and reconstruction parameters at our center include: scan coverage-entire thoracic aorta (lung apices to 2 cm below the celiac artery), contrast-intravenous injection of 95-mL iopamidol 370 mg I/mL at 4  mL/s followed by a 100-mL saline chaser at 4  mL/s, electrocardiographic-gating with axial reconstructions at 0.625-mm section thickness at 75% of the R–R cycle and 40% adaptive statistical iterative reconstruction. The selected scans were split the scans into five disjoint sets of about 42 scans each. We use five-fold cross validation for model evaluation where each fold uses four sets of CT scans for training the CNN, and one set for testing it. Manual aortic measurements were performed on 60 of the test scans at standard anatomic locations by three independent expert analysts, with the average value between raters used to define the ground truth measurements against which the output of the fully automated approach was compared. This retrospective study was performed as part of an Institutional Review Board, HIPAA-compliant study (HUM00133798) with a waiver of informed consent at the University of Michigan, Ann Arbor, Michigan.

### 2.3 CNN-Based Joint Segmentation and Localization

**Fig. 1:**
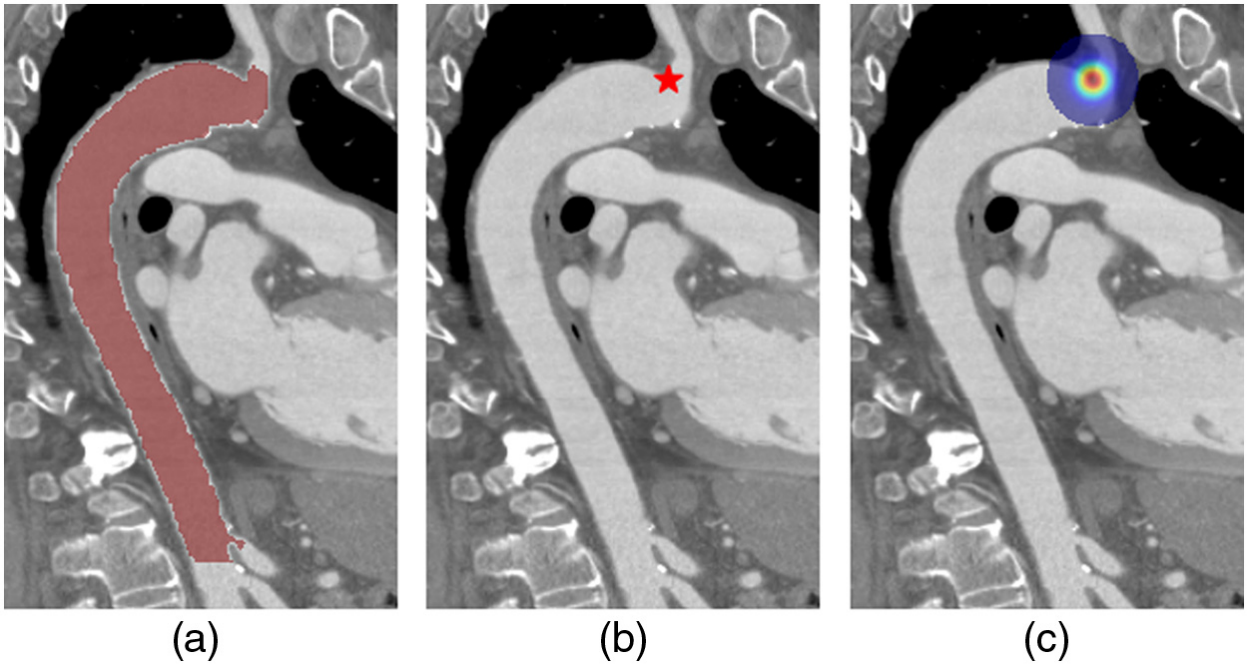
Example slice from a CT scan with its CNN targets. (a) Binary aorta segmentation mask (red), (b) just distal to left subclavian artery landmark (L3; red star), and (c) heatmap for learning L3 localization.

**Fig. 2:**
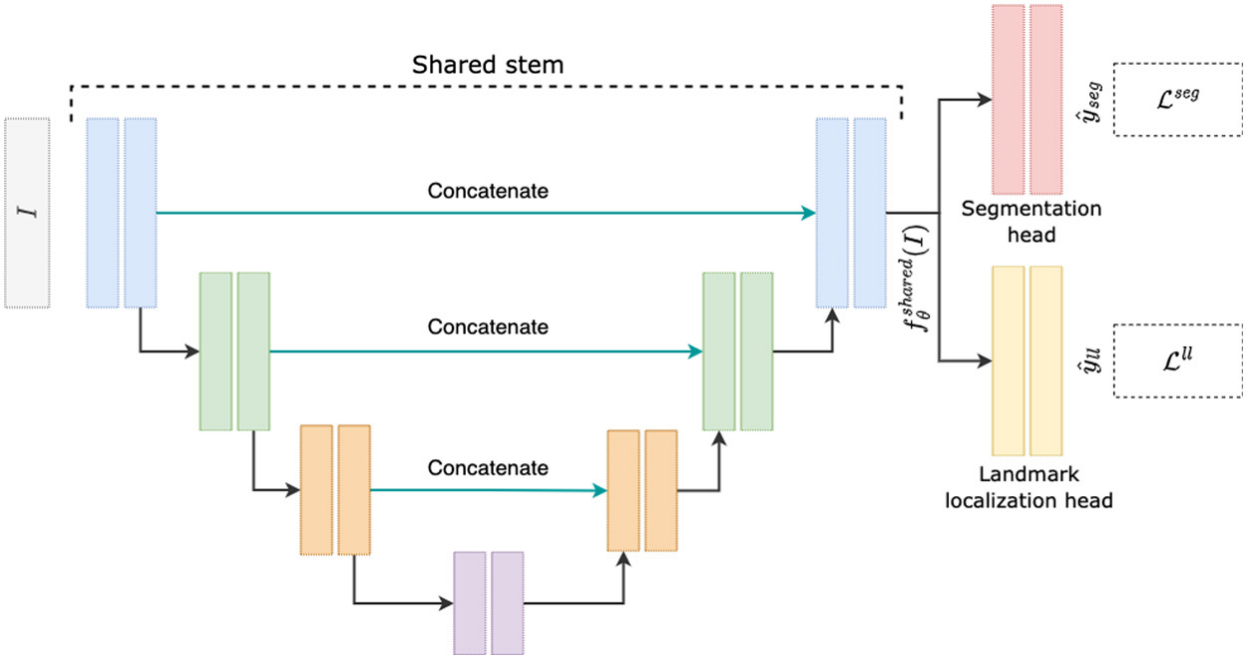
The architecture of the CNN used. We have a UNet stem that branches out into separate heads for each task, i.e., segmentation and landmark localization.

Our CNN is tasked to perform both segmentation and localization. The model’s input is a CTA scan, and its target outputs are a binary segmentation mask of the aorta and the coordinates of six anatomic landmarks, L1 to L6. Figure 1 shows an example of a segmentation mask and L3 landmark. The network utilizes a 3D UNet^14^ stem (with shared parameters θ) $f_{\theta }^{\text{shared}}$ and two separate branches $g_{\phi }^{\mathrm{seg}}$ and $g_{\psi }^{\mathrm{ll}}$ for segmentation and landmark localization, respectively (Fig. 2).

The CNN estimates the probability of each voxel being a point inside the aorta as $g_{\phi }^{\mathrm{seg}}(f_{\theta }^{\text{shared}}(I))\in [0,1{]}^{H\times W\times D}$. Similarly, $g_{\psi }^{\mathrm{ll}}(f_{\theta }^{\text{shared}}(I))\in [0,1{]}^{L\times H\times W\times D}$ is a vector such that $g_{\psi }^{\mathrm{ll}}(f_{\theta }^{\text{shared}}(I))(l,h,w,d)$ contains the probability that the voxel at (h,w,d) contains the landmark indexed by l.

Landmark localization is learned via heatmap regression.^15^ For each landmark, a 3D Gaussian heatmap is generated with its center at the landmark’s coordinate; the CNN learns from this heatmap. Instead of using the MSE loss typically used for heatmap regression, we use a weighted cross-entropy (WCE) loss that was found to stabilize training and improve accuracy.^13^ Given ground truth landmarks $L_{l}^{*}\in R^{3}$ for each landmark l in a given image I, the landmark localization loss is calculated as

Lll=WCE(y^ll,yll;α)=−1LHWD∑l,h,w,d[αyll(l,h,w,d)log(y^ll(l,h,w,d))+(1−α)(1−yll(l,h,w,d))log(1−y^ll(l,h,w,d))], (1)

where $y^{ll}(l,h,w,d)=\exp(\frac{{\left\|[h,w,d{]}^{T}-L_{l}^{*}\right\|}_{2}^{2}}{2\sigma^{2}})/\exp(-2\sigma^{2})$ is a normalized heatmap voxel for landmark l and y^ll=gψll(fθshared(I)) is the UNet heatmap estimate. In order to prevent the network from collapsing into the trivial all-zero local minimum solution and to promote predictions of positive values, we employ a high alpha value (>0.5) in the loss function.

Binary cross-entropy was used for segmentation loss $L^{\mathrm{seg}}$. Thus, the total loss optimized by the network is $L^{\mathrm{seg}}+\lambda L^{ll}$ where λ balances the contribution of each of the two losses. In all, we set the loss parameters to σ=5, λ=10, and α=0.9 for training.

Data augmentations can improve a model’s generalization performance when training on limited datasets by increasing the number of unique inputs the CNN is trained on.^16^ Thus, we train our network with randomly cropped CT images augmented by performing small random rotations. To perform a random crop, a voxel is randomly selected as the center and then the CT image is cropped around that voxel to a size of 96×96×160  voxels. A small random rotation is then applied to the cropped CT, which serves as a training sample to the CNN.

### 2.4 Post-Processing

Following inference, the CNN’s segmentation output is binarized by setting voxels with segmentation probability greater than 0.5 to 1 and the rest to 0. The final aorta segmentation was taken as the largest connected component from this segmentation output. The predicted coordinate for landmark l is the voxel from the UNet’s landmark output for l with the highest value.

### 2.5 Aortic Measurements

The final aorta segmentation and six landmark coordinates from the CNN are used to automatically perform diameter measurements using the following part of the pipeline.

#### 2.5.1 Centerline generation

To determine the centerline of the aorta, the aorta was first skeletonized.^17^ The skeleton often has branches in the aortic root and branch vessels. To reduce this to a single branch, we first found points on the skeleton nearest to the STJ and celiac landmarks. Next, the original skeleton was converted to its connected graph representation. Finally, shortest path between the STJ and celiac points was found using Dijkstra’s algorithm.^18^ The resulting endpoints and the shortest path between them become the final centerline.

#### 2.5.2 Aortic cross-section localization

The center of each cross-section at the STJ, proximal arch, mid-arch, and celiac was determined by finding the centerline point nearest to the corresponding landmark point inferred from the CNN. For the measurement at 2 cm distal to left subclavian, we find the closest centerline point to the left subclavian landmark and move 2 cm distal along the centerline. We perform a similar measurement 2 cm proximal to celiac artery landmark. The half-way point along the centerline between the STJ (L6) and proximal arch (L2) defines the location for mid ascending measurements. Similarly, the mid-descending measurement is obtained by moving along the centerline to halfway between the left subclavian (L3) and celiac (L4) arteries.

The vector normal to each cross-section was estimated as the difference between the coordinates of the two neighboring centerline points.

#### 2.5.3 Sinuses of Valsalva measurement

The sinuses of valsalva (SVS) are the widest part of the aortic root and represent the first aortic segment beyond the aortic valve (L1). To obtain the SVS measurement, we considered all the centerline points between the aortic annulus and the STJ. The maximal diameters at each of these points were considered and the highest was reported as the SVS measurement. In some cases, the cross-sections can include one or both of the coronary arteries, leading to overestimation of diameter measurements. To alleviate this issue, we employed a technique to remove the proximal coronary arteries from the SVS cross-section. First, fit a cubic spline to the boundary of the plane and estimated the curvature at various points on the boundary, which are indicative of branch vessels. We then removed the five points with the highest curvature from original boundary and found a new boundary by fitting a linear spline on the remaining points.

## 3 Results

### 3.1 Single versus Multitask Training

Multi-task learning has been shown to improve generalization of deep-neural networks including in our prior work on this application.^9^^,^^19^ To test this for our expanded dataset, we perform a fivefold cross validation experiment comparing our network trained for joint segmentation and localization (multitask network) against one trained exclusively for segmentation and one trained exclusively for localization (single task network).

**Fig. 3:**
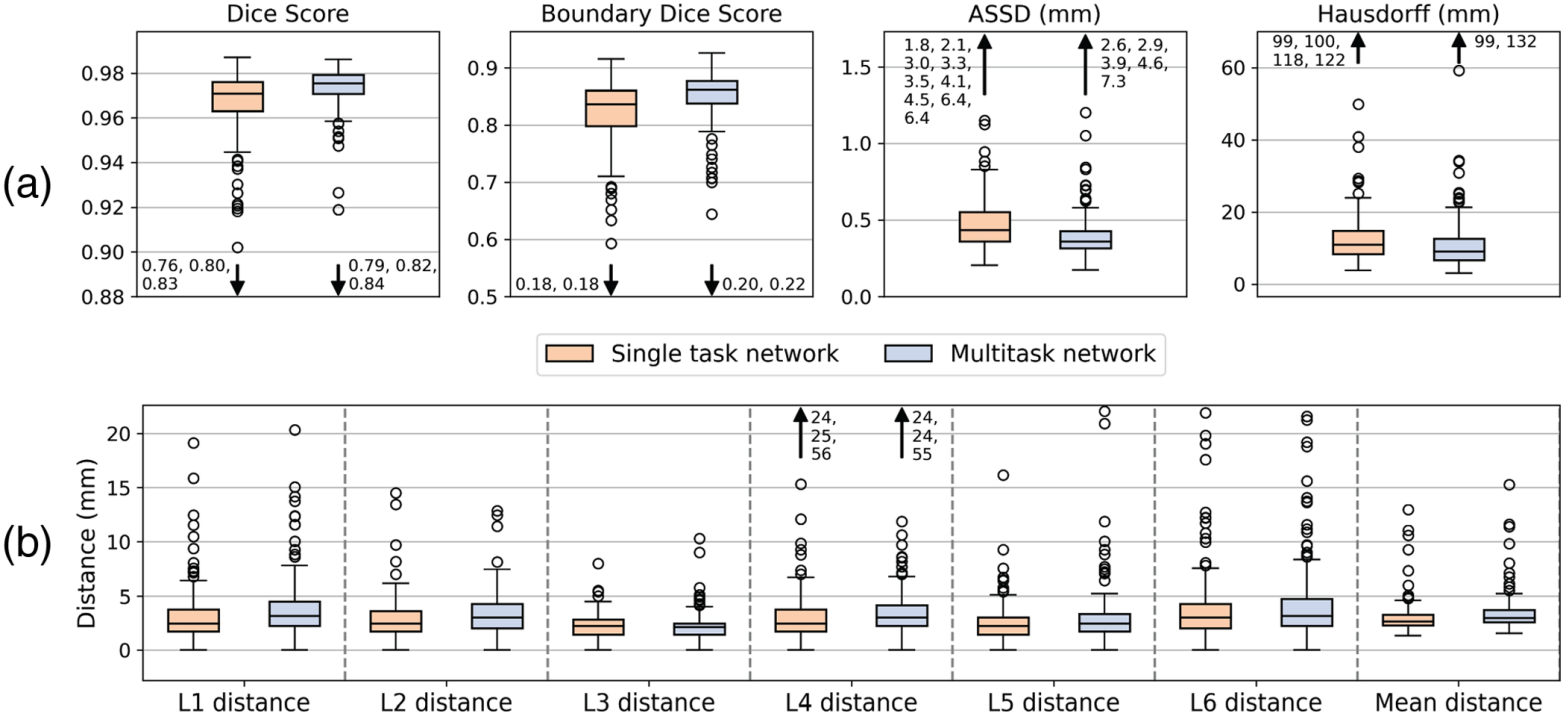
Single versus multitask network performance on (a) segmentation and (b) landmark localization. The numbers adjacent to arrows denote the location of extreme outliers.

Dice score, average symmetric surface distance (ASSD), and Hausdorff distance are used to measure segmentation performance. We also derive the boundary of the prediction and target segmentations and report the Dice score between the two as “boundary Dice score.” Landmark localization performance is assessed with Euclidean distance between predicted and labeled landmark coordinate. Higher is better for Dice score while lower is better for all other metrics. Figure 3 shows box plots of the results for the single task and multitask approaches. Visually, the two approaches have similar performance with the multitask network performing better segmentation and the single task network performing better landmark localization.

Tables 1 and 2 give the mean, standard deviation, paired t-test result for each evaluation metric. Overall, the multitask network performs statistically better segmentation across all metrics (p<0.0002) including boundary Dice, which is especially important for diameter measurements. The single task network performs better landmark localization (p<0.0452) across all landmarks except L3 (p>0.85). Given that segmentation is the more important task for determining accurate aorta diameter measurements, and considering that landmark distances were small (<1  mm in the 95% confidence interval), we opt to use the multitask network as the CNN for evaluating our pipeline against manual raters.

**Table 1:** Single task and multitask network segmentation performance (mean ± std) and paired t-test results.

Network or statistic	Dice	Boundary Dice	ASSD (mm)	Hausdorff (mm)
Single task	96.6±2.4	82.0±8.0	0.61±0.78	14.2±14.8
Multitask	97.2±2.1	84.7±7.4	0.48±0.65	11.6±12.0
p-value	<0.0001	<0.0001	<0.0002	<0.0002
95% confidence interval	[0.3, 0.9]	[1.7, 3.7]	[−0.21, −0.05]	[−4.1, −1.2]

**Table 2:** Single task and multitask network landmark localization performance (mean ± std.) and paired t-test results. Refer to Fig. 5 for visualization of L1 to L6.

Network or statistic	Euclidean distance (mm)						
	L1	L2	L3	L4	L5	L6	Mean
Single task	3.2±2.4	2.9±1.7	2.2±1.1	3.4±4.6	2.4±1.6	3.6±3.1	2.9±1.4
Multitask	3.8±2.7	3.3±1.9	2.2±1.3	3.8±4.4	2.9±2.5	4.0±3.4	3.3±1.6
p-value	<0.0001	<0.0029	>0.85	<0.0452	<0.0174	<0.0070	<0.0001
95% conf. interval	[0.3, 0.9]	[0.1, 0.7]	[−0.2, 0.2]	[0.0, 0.7]	[0.1, 0.8]	[0.1, 0.7]	[0.2, 0.5]

The results in Fig. 3(b) and Table 2 contrast with our prior work on this application,^9^ which showed that the multitask network was also more accurate for landmark localization. The benefits of multitask training can diminish when larger datasets are used, which may partly explain this difference in conclusion. Specifically, our new dataset has two additional landmarks that can enable the single task CNN to learn more general features.

### 3.2 Comparison of Automated Measurements Against Human Raters

**Fig. 4:**
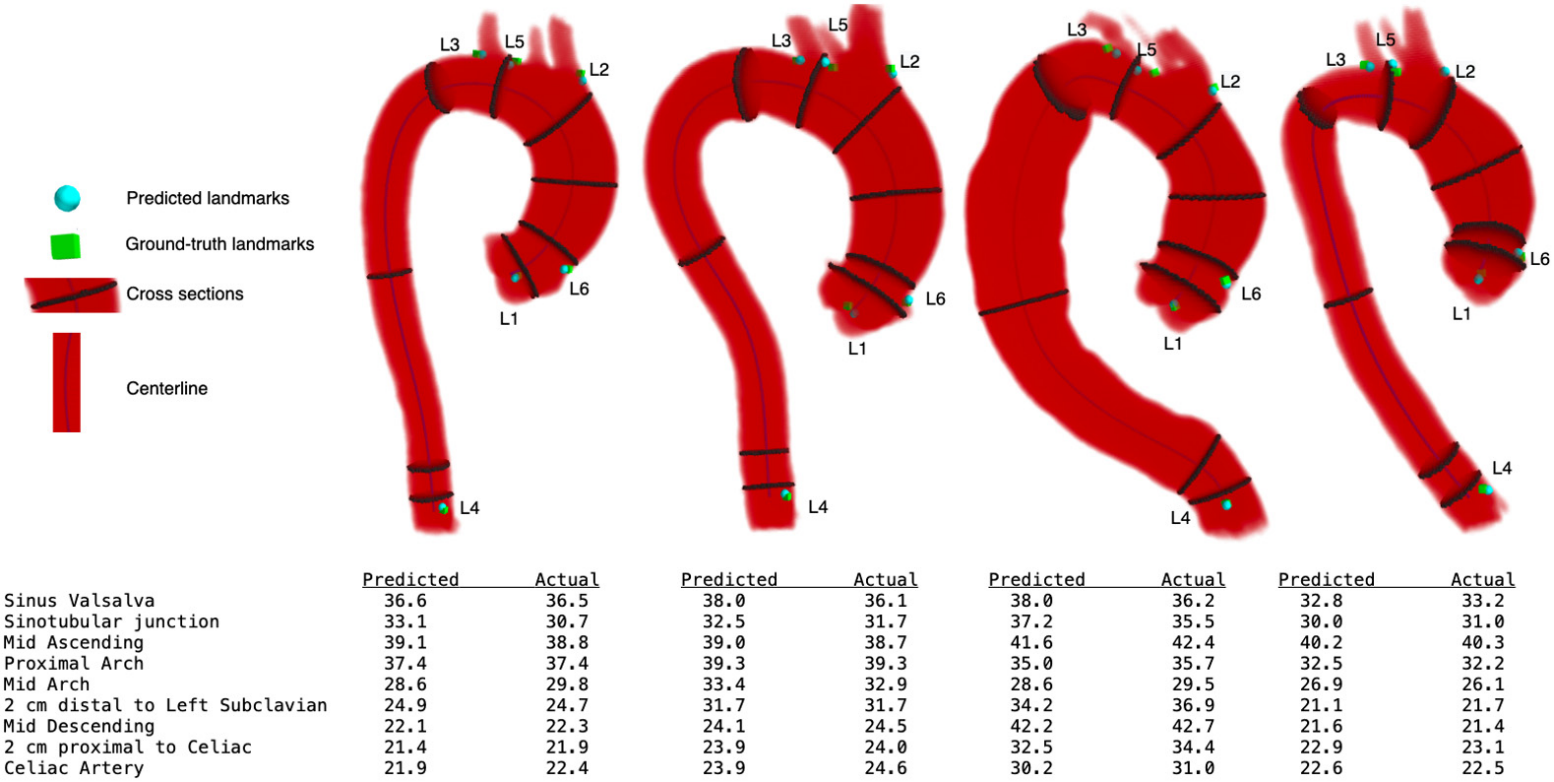
Representative cases demonstrating predicted segmentation, ground-truth and predicted landmarks, aortic centerline, cross-sectional measurement planes, and extracted diameter predicted and actual diameter measurements. We demonstrate that our automated approach yields accurate landmark localization and aortic diameter measurements across a range of aortic geometries including: mild ascending dilation (left), mild ascending dilation with tortuosity and bovine arch (center left), arch and descending dilation with tortuosity (center right), and mild ascending dilation with acute arch angulation and bovine arch (right).

We compared the aortic measurements obtained via our method against the average measurements from three raters. It was observed that the mean absolute difference between measurements were ≤1  mm at six of nine aortic locations but ranged from 1.43 to 2.18 mm at the SVS, STJ, and mid arch locations, as shown in Table 3. Mean absolute error was highest at the SVS (1.96 mm), STJ (2.18 mm), and mid arch (1.43 mm), although the standard deviation of manual rater measurements was also highest in these locations (1.20, 1.77, and 1.17 mm, respectively). Bias was <1.5  mm at all locations, with our automated approach demonstrating a small degree of systematic over-measurement in the SVS and STJ, whereas in all other aortic locations the automated approach slightly overestimated aortic measurements relative to manual raters. Representative examples of segmentation, landmark localization, and measurement planes are visualized in Fig. 4.

**Table 3:** Comparison of the diameter measurements from ML method versus average measurements from three expert raters by aortic location. The first column reports the mean absolute error between automated and manual diameters. The second column reports the average difference (bias) between rater-measured and algorithm-output diameters (actual minus predicted). The third column reports the 95th percentile range of the measurement error. The fourth column reports the average standard deviation in manual diameter measurement for comparison.

Location	Mean abs. error (mm)	Bias (mm)	95 %ile range (mm)	Rater std. dev. (mm)
SVS	1.96	−1.38	[−5.90, 3.14]	1.20
STJ	2.18	−0.55	[−5.97, 4.87]	1.77
Mid ascending	0.56	−0.21	[−1.57, 1.14]	0.65
Proximal arch	0.70	0.40	[−1.30, 2.10]	1.17
Mid arch	1.43	0.35	[−4.44, 5.13]	1.19
2 cm distal to left subclavian	0.63	0.24	[−1.56, 2.04]	1.01
Mid descending	0.75	0.08	[−2.80, 2.96]	0.97
2 cm proximal to celiac	0.65	0.36	[−1.54, 2.26]	0.86
Celiac artery	0.67	−0.04	[−2.05, 1.97]	1.13

**Fig. 5:**
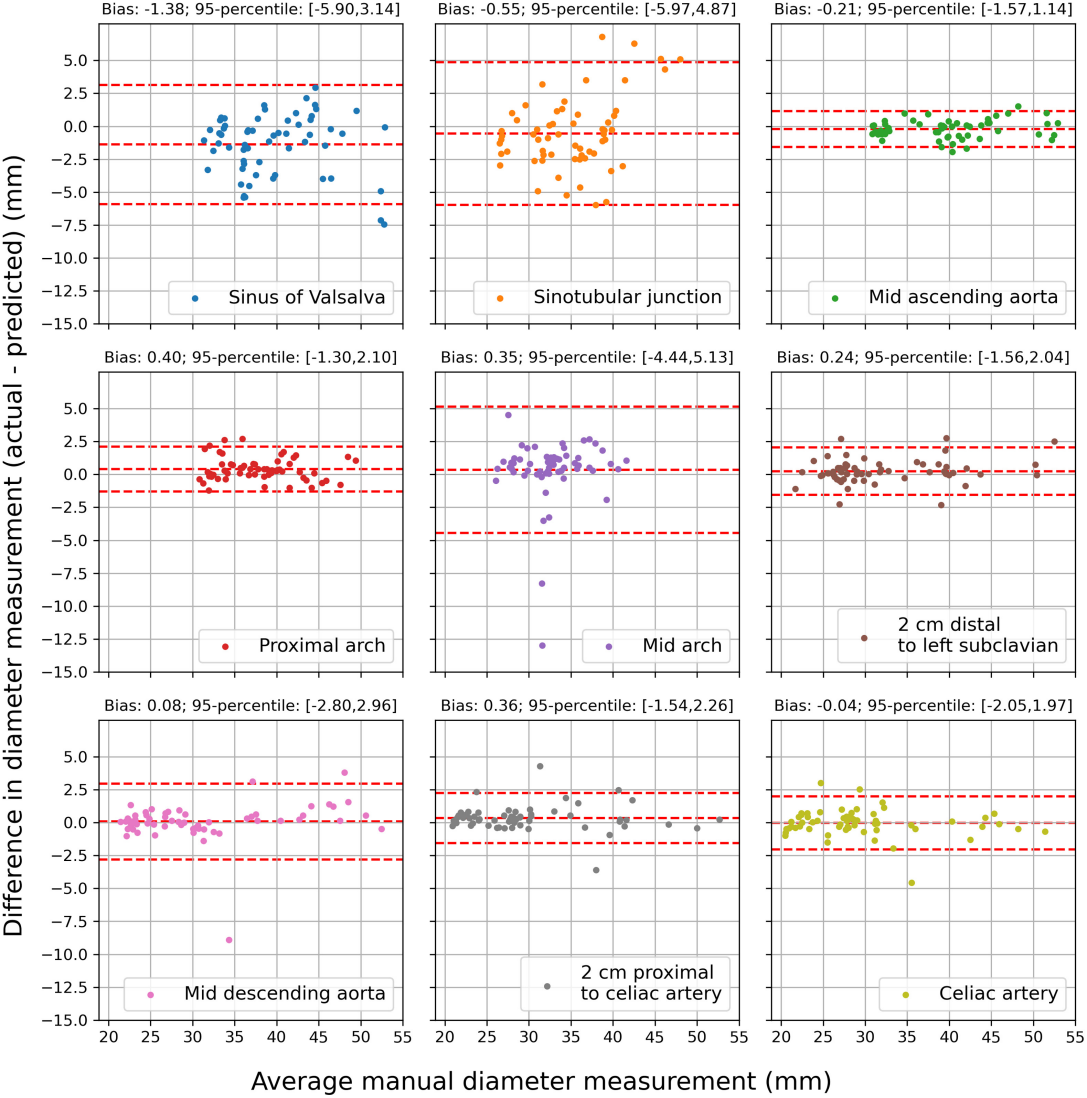
Bland–Altman plots showing the difference between actual (average of three expert raters) and predicted diameter measurements for at all nine standard aortic measurement locations.

Bland–Altman plots showing the difference between the gold-standard manual diameter measurements (average of three raters) and automated diameter measurements for all landmarks are shown in Fig. 5. Limits of agreement were smallest in the mid ascending aorta (−1.57 to 1.14 mm), a common location of the maximal aneurysmal dimension thus one of the most clinically important anatomic locations. Conversely, the limits of agreement were highest at the SVS (−5.90 to 3.14) and mid arch (−4.44 to 5.13).

We note that the average time taken by the rater is 24 min (range: 15 to 38 min), whereas our method takes about 15 s per CTA scan on a standard high-performance laptop with a GPU.

### 3.3 Effect of Sinus Valsalva Correction

**Fig. 6:**
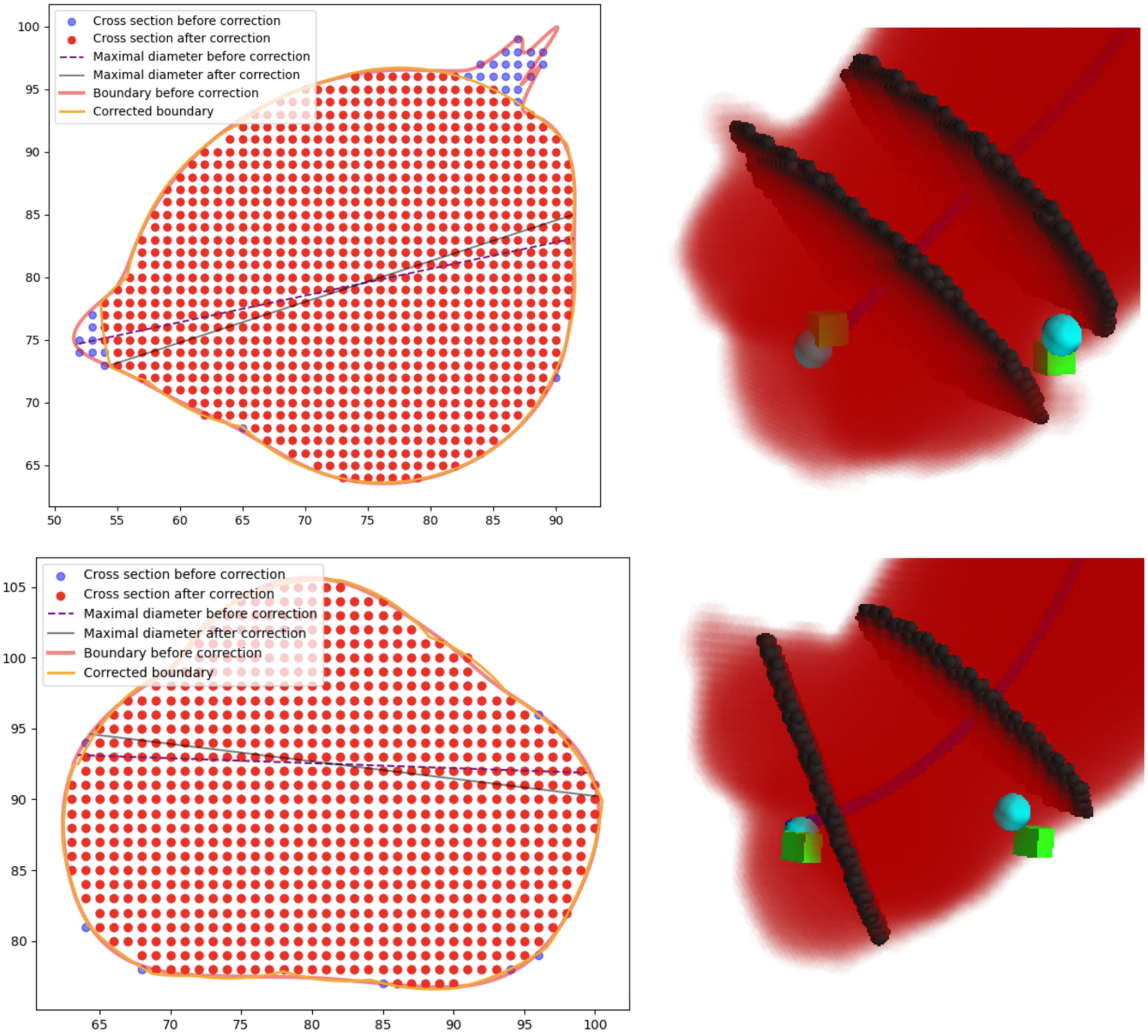
Visualization of the results of our cubic spline process refining the aortic boundary at the SVS (as described in Sec. 2.3.3). We demonstrate that correction happens only when branching coronary arteries are present in the selected aortic cross-section (top left) and not when absent (bottom left). Corresponding segmentation results of the aortic root with predicted (teal sphere) and ground-truth (green cube) L1 and L6 landmarks are also shown (right).

We observe that the mean absolute error reduces from 2.57 to 1.96 mm after including the SVS correction process. A visualization of the procedure of correction and its effects is shown in Fig. 6.

## 4 Conclusion

**Fig. 7:**
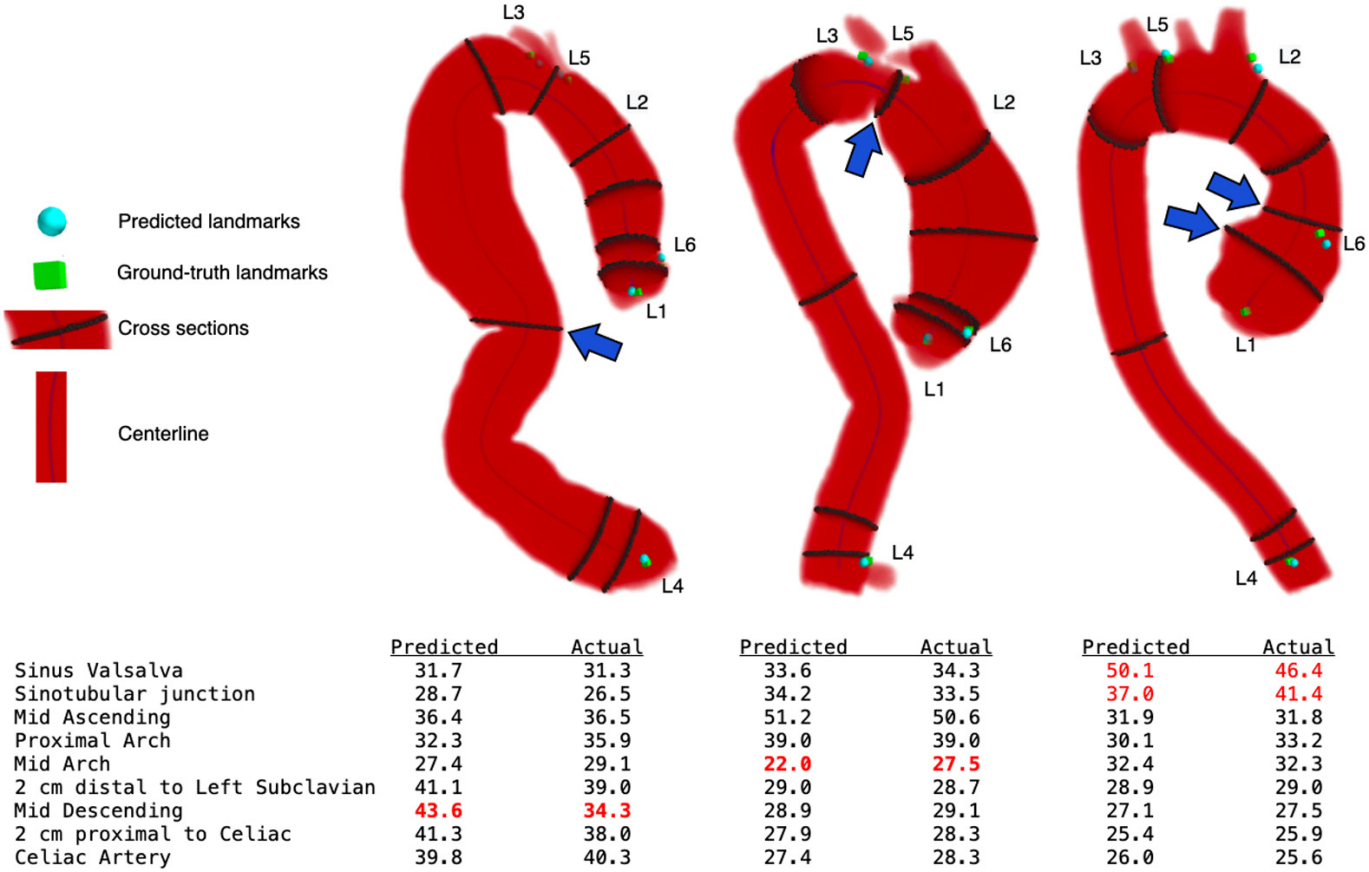
Representative cases where there was substantial disagreement between predicted measurements and actual measurements from expert raters at a specific aortic location (blue arrow). Disagreements tended to occur in aortic segments with irregular anatomy characterized by tortuously and/or rapidly changing diameter along the centerline. Shown is a case with a 9.3 mm discrepancy at the mid-descending level (left), a case with a 5.5 mm discrepancy at the mid-arch (middle), and a case with up to 4.4 mm discrepancy at the SVS and STJ (right).

We demonstrate a simple and rapid ML-based method to obtain automated aortic measurements using a CNN trained for segmentation and landmark localization and show that added accuracy is attained by joint training a single CNN to perform both tasks simultaneously. The obtained diameter measurements errors were within 1 mm of human reported diameters at the majority of the nine locations analyzed, although errors were higher (1.4 to 2.2 mm) at the SVS, STJ, and mid arch, locations that are characterized by more irregular aortic contours and rapid changes in diameter over short lengths (Fig. 7). It is important to note that the variance of expert manual rater measurements was also the highest at these locations, supporting prior reports suggesting that these anatomic locations are the more challenging to reproducibly measure due to their anatomy.^20^

The first part of our pipeline uses a 3D UNet to segment the aorta and localize landmarks. We achieve a better Dice score (97±2.1) and ASSD (0.61±0.78  mm) compared to other works that use a 2D UNet (Dice: 91.22±8.97; ASSD: 4.46±4.54  mm).^12^ Our median Dice score (97.5) and ASSD (0.36 mm) were better than the median Dice (95) and ASSD (0.99 mm) of another 3D UNet approach,^6^ but our median Hausdorff was a bit worse (9.11 versus 8.00 mm). Direct comparisons between studies is, however, limited by important differences in datasets such as heterogeneity of aorta pathology studied. In all, the accuracy of our 3D UNet was found to be within the desired tolerance for accurate diameter measurements, which is the focus of our application.

When compared to a similar, recently described deep learning pipeline for automated aortic measurement, our pipeline performed substantially better (e.g., 95th percentile errors of about 6 mm in our study compared to 10 mm in prior reports^7^^,^^8^). However, these prior reports evaluated a more heterogeneous patient population including pathologies other than aortic aneurysm (e.g., aortic dissection), which can dramatically alter aortic geometry and contrast levels. Other works on automated diameter measurement are not fully automated or focus on a subset of the nine clinically standard locations.^6^^,^^10^ Given appropriate training data, our diameter measurement pipeline has the potential to generalize to other patient populations like those with abdominal aneurysm or with dissections and to other scan types like MRI.

The results of our work have potential practical applications for daily clinical analysis. First, the fully automated process we describe in this work could obviate the need for significant human effort spent performing the tedious and repetitive task of standardized aortic measurement. In clinical practice we expect that some human interaction will still be required to verify the results of automated analysis and correct any erroneous measurements. However, the substantial time savings of an automated approach (i.e., 15 s compared to at least 15 min) promises to allow substantial human effort to be re-distributed to higher-level diagnostic tasks, a potentially impactful change given increasing productivity demands and rates of burnout in diagnostic radiology.^21^^,^^22^ Furthermore, many smaller hospitals do not have resources to support aortic measurement work in a dedicated 3D laboratory, and thus an automated solution such as the one we describe may facilitate broader availability of high-quality and comprehensive aortic measurements for patients with TAA.

In conclusion, we present a fully automated pipeline for accurate and comprehensive thoracic aortic measurement in patients with TAA using CTA data and a joint localization and segmentation CNN. Our automated pipeline results in significant time saving for aortic diameter analysis, with measurement results that are comparable to that of expert manual raters in most instances. However, measurement accuracy remains suboptimal at regions highly variable aortic anatomy, such as the SVS and mid-arch, although these regions are also challenging for human raters. Future effort will focus on further refining our automated processes to address current inaccuracies as well as expanding the applicability our pipeline to non-gated, and potentially non-contrast CTA imaging, as well as more diverse types of aortic pathology.

## Data Availability

The CT data used to train the models in this paper are derived from clinical data from our hospital and are not available for public sharing based on University policy. The code used for the experiments described in this paper is available at GitHub: https://github.com/Burris-Group/Fully-Automated-Aorta-Measurement-Pipeline.
